# Possibilities for Maintaining Appetite in Recovering COVID-19 Patients

**DOI:** 10.3390/foods10020464

**Published:** 2021-02-20

**Authors:** Alexander Teymour Zadeh Baboli Høier, Nora Chaaban, Barbara Vad Andersen

**Affiliations:** Food Quality Perception and Society, Department of Food Science, Aarhus University, Agro Food Park 48, DK-8200 Aarhus N, Denmark; alexanderhoier@gmail.com (A.T.Z.B.H.); ncho_on@hotmail.com (N.C.)

**Keywords:** COVID-19, sensory function, chemosensory dysfunction, perception, appetite, well-being, pleasure, recovery, interview

## Abstract

COVID-19 and sequelae thereof are known to cause chemosensory dysfunction, posing a risk for intake and adequate nutrition for recovery. The overall objective of this study was to investigate the subjective strategies for maintaining appetite applied by patients recovering from COVID-19. The study included 19 in-depth interviews, focusing on patients suffering from long-term effects of COVID-19. The results were analysed using a thematic analysis for qualitative data. Results on strategies for maintaining appetite included four key themes: (1) a focus on well-functioning senses, (2) a focus on familiar foods, (3) a focus on the eating environment, and (4) a focus on post-ingestive well-being. It was found that factors prior to, during and after food intake, as well as the context, could influence desire to eat and pleasure related to food intake. As ageusia and anosmia make characterization of food difficult, being able to recognize and memorize its flavour was important to engage in consumption. Under normal circumstances, the hedonic value of food relies predominantly on the flavour of foods. When suffering from chemosensory dysfunction, shifting focus towards the texture of food, including trigeminal stimulation during consumption, were beneficial for maintaining appetite and food-related pleasure. Furthermore, a focus on the holistic satisfying feelings of choosing healthy food, as well as a focus on other people’s enjoyment during meals were reported to boost well-being around food intake. The study elaborated our understanding of the complex consequences of COVID-19, and can be applied in health promoting initiatives targeted patients recovering from COVID-19.

## 1. Introduction

Severe acute respiratory syndrome coronavirus 2 (SARS-CoV-2), also referred to as ‘coronavirus disease’ (COVID-19), has spread rapidly all around the world. Besides a mortality rate of COVID-19 on 2.2% as of the 8th of February 2021 [1], the long-term effects can be devastating on subjective quality of life. The long-term effects of COVID-19 vary from patient to patient, but often include chemosensory dysfunction in terms of dysosmia and dysgeusia [2]. Dysosmia is a condition affecting smell perception, and can be broadened out to the conditions; ‘anosmia’, which is a complete loss of the ability to detect odours, ‘parosmia’, which alters the odour perception, often to displeasing odours, ‘hyposmia’, which is decreased ability to detect odours, and ‘phantosmia’, which concerns spontaneously occurring odours without any triggers [3]. Dysgeusia is a condition concerning alterations of the perception of basic taste. This condition can be broadened out to; ‘ageusia’, which is a total loss of the ability to taste and ‘parageusia’, which alters the taste perception, often to displeasing tastes, and can be triggered by any or specific tastes [3].

Vaira et al. [2] found that as many as 85% of COVID-19 patients suffered from chemosensory dysfunctions in the beginning of the acute phase. Approximately 50% of the patients showed chemosensory dysfunctions two to three weeks after infection [4], and after 60 days, 7.2% were found to suffer from chemosensory dysfunction [2].

As malnourishment for an extended period of time weakens the body’s immune system, proper nutrition is important for the recovery of disease [5]. Likewise, proper nutrition is believed to be important for the recovery of COVID-19, and not least for subjective well-being. The food’s sensory properties, and thereby the perception of odours, flavours, and basic tastes, are the main factors driving human motivation to eat and intake [6]. The importance of sensory properties for hedonic perception of foods and food behaviour is evident when observing the broad range of studies and models focusing on: sensory properties and acceptance, e.g., [7,8,9], sensory properties and preference [10], sensory properties and food behaviour [11,12,13,14], and liking as a determinant for intake [15]. A dysfunctional sensory perception therefore poses a serious risk to appetite and the nutrition needed for a fast recovery. A study of the characteristics of patients with chemosensory disorders showed that 50% of participants changed their food habits and preferences [16]. Furthermore, chemosensory dysfunction poses a significant impact on day-to-day life [17]. Affected subjects report reductions in quality of life [18,19,20,21], with reduced pleasure from eating being one of the most distressing symptoms of chemosensory loss and the main complaint from patients seeking medical attention [16].

Sensory properties are, however, not the only drivers of motivation to eat, and therefore, insight into how COVID-19 patients cope with chemosensory dysfunction in order to maintain motivation to eat and food-related pleasure, can lead to actionable knowledge for use in dietary therapy supporting recovery, and further, to advise patients on how to maintain food-related quality of life in general.

The overall aim of the present study was to investigate the subjective strategies for maintaining appetite applied by patients recovering from COVID-19. Specifically, the objectives were to study how COVID-19 affected appetite, sensory perception and food-related pleasure, and further to study, how patients cope with chemosensory dysfunction to maintain appetite and food-related pleasure.

## 2. Materials and Methods

The study applied one-on-one in-depth interviews, due to the type of information required by the participants. In-depth interviews allow the participants to unfold any sensitive personal matter that would not have been appropriate to discuss in a group [22], and further, this method allows the researcher to collect detailed information beyond the surface level. Up until now, the study of strategies for coping with changes in appetite due to COVID-19 has been an unexplored field of research. Therefore, a semi-structured interview guide was chosen to ensure that all predefined themes were addressed; also, the semi-structured design allowed the researcher to follow up new points raised by the participant that had not been thought of when preparing the interview guide.

Prior to data collection, the Central Denmark Region Committees on Health Research Ethics approved the study being conducted. The participants were informed that the interview was being recorded, and were also informed of their legal rights, and how their data would be used and stored. The participants gave verbal consent for the use of their replies in the research study. The interviews were conducted using Zoom or similar communication platforms depending on the preference of the participant. Telephone interviews were conducted in a few cases, when the participants were unable to partake in a video interview. The interviews were recorded using the record function in the video meeting application, or using a dedicated telephone recording application. Video interviews were preferred, in order to get a better understanding of the participants, and enabling their body language to be read.

### 2.1. Participants

A total of 19 interviews were conducted among a Danish population suffering from long-term effects of COVID-19, meaning that participants were in the post-acute phase of Codid-19, yet were still showing symptoms, including changes in appetite. Participants were recruited via posts in a Danish Facebook group for people suffering from long-term effects of COVID-19. Inclusion criteria were: being over 18 years of age and suffering from long-term effects of COVID-19 including changes in appetite. All participants reported being in the post-acute phase of COVID-19 at the time of data collection. A total of 14 participants had been diagnosed with COVID-19 through antibody or swab test. The remaining five participants had been diagnosed with COVID-19 via subjective assessment, due to not being eligible for a test through the Danish health care system at the time of the disease. These five participants all showed regular long-term effects of COVID-19 symptoms, such as anosmia, ageusia, and fatigue. Across the total study sample, the average period for showing symptoms during the acute phase of the disease was reported to be 16 days, and none had been hospitalized due to COVID-19. The most frequent symptoms during the acute phase of COVID-19 included fever, ageusia, fatigue, anosmia, throat pains, headache, and difficulties breathing. In the post-acute phase, the most frequent symptoms included ageusia, anosmia, fatigue, parosmia and difficulties breathing. Other less common symptoms included headache, feeling nausea, difficulties concentrating, throat pains, parageusia and phantosmia. One participant (participant #2) reported having suffered from a blood clot earlier in life. As the present study focused on strategies for coping with changes in appetite due to COVID-19, and the blood clot incident had been years earlier in the participant’s life, it was decided to include the participant in the present study. The remaining participants had not been diagnosed with diseases other than COVID-19. Participant characteristics can be seen in Table 1.

### 2.2. Pilot Test

The interview guide was pilot-tested among four colleagues and naive participants with different ages, educational backgrounds and COVID-19 histories; one of the participants had been diagnosed with COVID-19, and the remaining three were non-diagnosed. None of the pilot participants were included in the final sample population. A pilot test is generally a recommended procedure when conducting interviews [23]. The pilot test aimed to ensure a proper flow and understanding of the questions, and to decide whether questions should be added and/or excluded. The interview guide was refined according to the feedback; questions were rephrased and words were replaced with ones that were more easily understood by all naïve participants.

### 2.3. Interview Protocol

The interviews lasted 45 to 90 min each and were conducted by two interviewers independently. Both had educational training in the qualitative research method. The interviewers developed the interview guide together, discussed the interview approach prior to conducting the interviews, and conducted the pilot test together. During the interviews, the interviewers followed the structure of the interview guide, the format of the questions, and discussed the interview style, in order to ensure uniformity and transparency in the interviewing style.

The interviews followed a semi-structured interview guide, a format that allowed the interviewer to pursue topics raised during the interview [24]. The interviewer firstly informed the participant about the purpose of the study. The following questions were designed to start the conversation and dialogue, including introductory, opening, transition, key and ending questions. The questions led the participants to reflect on health, appetite, sensory perception ability and food-related pleasure. It was emphasized that the questions were easy to understand, short, clear and could engage the participants in detailed elaboration. To explore the strategies affecting participants’ appetite, they were firstly directly asked about how they coped with changes in appetite, sensory perception ability and food-related pleasure. If the participant reported not having consciously applied any strategies, they were asked to elaborate on concrete situations regarding every theme presented in the interview guide. This allowed the researcher to analyse for coping strategies applied subconsciously. All participants were interviewed once during the study, and at the end of the interview, the participants were thanked for their participation and assured anonymity.

### 2.4. Data Analysis

Researcher triangulation was conducted in order to ensure all important points from the interviews were included in a thematic analysis—the foundational analytical method. Researcher triangulation is generally recommended to overcome fundamental biases, arising when using a single researcher [25]. Different types of researcher triangulation can be applied: data triangulation, method triangulation, investigator triangulation and theory triangulation, respectively. In the current study, investigator triangulation was applied by using more than one researcher to conduct the interviews and analyse the data. Prior to the thematic analysis, the two interviewers independently and without prior discussion conducted descriptive summaries of all the interviews. The descriptive summaries served to capture a summed picture of each interview, and as such, provided the basis for the thematic analysis, together with the video material. From the summaries, the two researchers, likewise without prior discussion, clarified the themes to address the overall aim of the study. Approximately 21 h of video material and the descriptive summaries created the basis for the thematic analysis. The investigator triangulation approach ensured that the same data set was interpreted by more than one researcher; each provided their independent analysis before further comparison, which is important for decreasing bias in the analysis of data [25].

**Figure 1:**
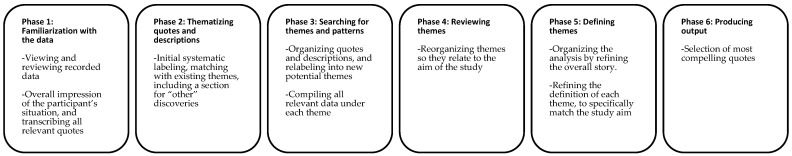
Overview of the phases conducted in the thematic analysis. Guidelines and adaptation from [26].

Thematic analysis is a method for identifying, analysing, interpreting and reporting patterns and themes within qualitative data [26]. The analysis included a systematic flow of moving forwards and backwards between the phases seen in Figure 1. The analysis progressed from familiarization with the data to a systematic change and reorganization of themes. The themes and concepts emerged from patterns in quotes and the descriptive summaries of the interviews. Patterns in responses and meanings related to the study aim were reflected in the chosen themes.

In case the verbal language did not clearly communicate a participants’ feeling or intention, and the feeling or intention was found to be important for the data interpretation, participants’ body language was taken into consideration. For example, if the liking of food was mentioned to be altered, but the participant did not describe the direction of the change clearly, (dis)liking could be revealed via facial expressions. Body language was mentioned as a note in the descriptive summaries and/or put in brackets after a quote. The thematic analysis was split into a descriptive part and an action-based part. Phase one and two, in Figure 1, were conducted independently, and afterwards combined and discussed in order to exclude any misinterpretations and to avoid any important points being missed. Afterwards, the two researchers conducted a thematic analysis for one part each. Towards the end of the analysis, the parts were combined and discussed, to ensure uniformity in the interpretation of the data.

## 3. Results

In this section, the results are divided into two sections; the first section describes the general subjective experiences of the effects of COVID-19 on appetite, sensory perception of taste, flavour and smell, and food-related pleasure, and the second section describes the subjective strategies applied by the participants to maintain their appetite and food-related pleasure. The latter section is based on the main themes that arose from the thematic analysis to maintain appetite: (1) focus on well-functioning senses, (2) focus on familiarity, (3) focus on well-being, and (4) focus on eating environment.

### 3.1. The Effect of COVID-19 on Appetite, Sensory Perception and Food-Related Pleasure

#### 3.1.1. Appetite

An effect of COVID-19 on appetite was mentioned by most participants. During the acute phase of COVID-19, most participants experienced a decreased appetite:

*Participant 7: Female, 59: “I had to convince myself to eat, and that was extremely difficult. The food had to be placed in front of me, and sometimes I ate, and at other times, I barely touched it.”*

*Participant 10: Female, 35: “If I were to describe it in one word, then it would be >forced< eating.”*

*Participant 12: Female, 40 “We had to remind each other (to eat), my husband and I, the first couple of weeks, because he did not feel like eating as well.”*

Besides suffering from decreased appetite during the acute phase of COVID-19, some participants were still suffering from a decreased appetite as a long-term effect of COVID-19 (*n* = 13):

*Participant 5: F, 26: “I still have a reduced appetite, and I do not really feel hungry” … “I do not experience that rumbling in the stomach as before, and the feeling of satiation come earlier than usual because I do not feel hungry to begin with.”*

*Participant 7: F, 59: “I then started sensing that I was a bit hungry, but I did not know what I wanted (to eat). I did not feel like eating because I could not really taste or smell anything.”*

*Participant 8: F, 42: “I do not think about food until I am really hungry”*

*Participant 10: F, 35: “Some days I do not feel like eating at all.”*

Though many participants described that they in general did not feel hungry nor a desire to eat during COVID-19, or reached satiation faster during a meal, some participants expressed the post-meal satiety sensation as ‘unsatisfying’ (*n* = 3). Satiation was not only depending on a physical feeling of fullness but had to include a feeling of ‘pleased senses’ based on the perceived sensory properties of food in order to feel fully satisfied. As a result, some participants expressed exposing themselves to food continuously in the search for ‘sensory satisfaction’:

*Participant 19: F, 34: “When I ate something, I did not feel satisfied (caused by the lack ability to perceive the sensory properties of food). Therefore, I had a desire to eat shortly after, but I did not eat a lot of food, because I could not taste it anyway.”*

*Participant 16: M, 48: “The less I was able to taste, the more I ate of it. (…) It was like walking around searching for something that could quench the thirst, but I was not able to find it.”*

*Participant 22: F, 25: “You also eat to achieve the taste experience, and if it is not there, then it is like something is missing. For every bite you take, you are hoping to be able to taste something. I actually feel that more food is needed for me to feel satiated.”*

*Participant 15: F, 33: “especially when I could not perceive any flavours, it was very difficult to tell, when I felt satiated.”*

#### 3.1.2. Sensory Perception

##### Taste Perception

During the acute phase, several participants expressed experiencing ageusia, with a complete lack of basic taste perception [3].

*Participant 8: F, 42: “I could not taste anything, neither salt, sour, sweet, bitter (during the acute phase).”*

*Participant 18: M, 53: “Citrus fruits, like lemon and orange that normally are very sweet or very intense in taste, these were completely tasteless.”*

In the post-acute phase, many participants reported suffering from ageusia, with the majority experiencing hypogeusia, a decreased basic taste perception, as basic taste perception gradually returned.

*Participant 3: F, 29: “It is just thick and warm (about the sensory experience of coffee).”*

*Participant 6: F, 54: “It was very slow, and it started coming back gradually (about perceiving basic tastes).”*

During the period where basic taste perception gradually returned, the participants expressed a need for higher concentrations of basic taste attributes to be able to perceive them:

*Participant 10: F, 35: “If there is too much salt (in the food), then I am able to perceive it. If you put regular amounts, then I can’t perceive it. Not at all.”*

*Participant 12: F, 40: “It has to be extreme (amounts) before I can taste it. When I make rice, I tend to put too much salt in and the others (her family) do not like it.”*

Perception of taste did not necessarily return equally fast for all basic taste perceptions. Several participants experienced a higher sensitivity towards single basic taste attributes compared to others. Whenever a specific basic taste attribute was perceived in a meal, it was found to completely dominate the flavour experience:

*Participant 20: F, 46: “I can taste salt, because I use a lot of it. Much more than I used to. I can barely taste sourness. I can taste sweetness.”*

*Participant 3: F, 29: “If I ordered iced coffee, then it should not be too sweet, because then that was the only thing I could taste. It quickly became too sweet, and that’s the same with sour and saltiness.”*

*Participant 13: F, 43: “If something is pickled, it gets an extremely intense vinegar taste.”*

*Participant 14: F, 66: “If lemon is present in food, then everything tastes sour. Lemon (sourness) takes over (the taste perception).”*

The gradual return of basic taste perception was explicitly expressed by the participants, but the total time period suffering from ageusia varied between participants.

*Participant 3: F, 29: “It disappeared for a week or two, and then it came back (about perceiving the basic tastes).”*

*Participant 19: F, 34: “I was not able to perceive basic tastes in three weeks, then it started coming back gradually, but not completely.”*

*Participant 16: M, 48: “The first two months (basic taste) were completely gone, and then I started being able to perceive sourness, saltiness and sweetness.”*

##### Odour Perception

When reflecting on the acute phase, several participants expressed a partial or complete loss of odour perception, defined as anosmia [3], making them incapable of characterizing food items.

*Participant 13: F, 43: “I tried eating chocolate cake, but it was completely tasteless (because she could not perceive the flavour of chocolate)”*

*Participant 7: F, 59: “I cannot tell if it is a pear or an apple, a blackberry or a blueberry.”*

Others described not being able to perceive flavour at all, indicating a combined basic taste and odour impairment.

*Participant 10: F, 35: “The other day, I accidently made a very strong curry dressing (..) and it was like eating soft ice cream, that does not taste of anything.”*

*Participant 15: F, 33: “I thought of eating mackerel in tomato with my oatmeal in the morning because I could not taste anything.”*

*Participant 6: F, 54: “If you had me blind-folded, and you put food in my mouth, I would not be able to guess what food it was.”*

During the post-acute phase, the majority of participant experienced a partial or complete loss of odour perception:

*Participant 12: F, 40: “I have always been extremely sensitive towards scents. I could throw up by the least disgusting scent, and I do not feel like that anymore. It really has to be the extremes (to be able to smell it).”*

*Participant 5: F, 26: “I was able to taste the sweetness (in a strawberry cake), but I could not taste the flavour of the chocolate, the strawberry and the cream.”*

*Participant 9: F, 50: “If you can imagine eating ketchup. The only thing you can perceive is the sweetness.”*

*Participant 22: F, 25: “I cannot taste the difference between food. I cannot taste if it is a tomato or if it is a cucumber. I can only taste the saltiness and the sourness.”*

In a few cases, the participants suffered from hyperosmia during the post-acute phase, defined as hypersensitive sense of smell [3].

*Participant 9: F, 50: “I really like lilies, and I think they smell amazing. Sometimes I cannot handle the scent (about her sense of smell being hypersensitive).”*

*Participant 7: F, 59: “I am a nurse, so the smell of faeces and stuff like that make me sick. It was not like that before (because the smell is too intense).”*

*Participant 6: F, 54: “I made a dish with cabbage in it, that I normally liked, and suddenly I could not handle the smell of the cabbage (because the smell of cabbage was too intense).”*

Some participants further experienced parosmia and phantosmia during the post-acute phase, defined as altered odour perception and the experience of odours that are not actually present, respectively [3]. The most common off-flavours mentioned were soap, metallic, rotten, smoked, perfume and chemicals:

*Participant 5: F, 26: “Some scents smell different compared to before. For example, my perfume smells different than before.”*

*Participant 2: F, 44: “I was in bed in an isolated place, and I could smell Christmas cookies. There were not any Christmas cookies (when she was in isolation at the hospital).”*

*Participant 8: F, 42: “There was a day, were I wanted to eat a big, delicious steak. (…) The taste is like how rotten animals smell.”*

*Participant 10: F, 35: “If I eat a crunchy piece of bacon, then it tastes like soap.”*

*Participant 13: F, 43: “I love Pepsi Max. That’s my drug. It is actually terrible (that she cannot drink it anymore). I can cry about it. I know it is such a piddly thing, but now it tastes like perfume. It is like someone is spraying perfume directly in my mouth.”*

*Participant 13: F, 43: “Salted almonds, that I also love. When I tried eating them recently, they tasted like smoke. That smoke taste is absolutely disgusting.”*

*Participant 16: M, 48: “The taste of alcohol is very, very strongly chemical.”*

*Participant 18: M, 53: “Everything tasted like iron.”*

*Participant 20: F, 46: “When I eat jam, it tastes like soap.”*

##### Chemesthesis Perception

A few participants experienced an altered sense of touch during the post-acute phase of COVID-19. Specifically, they reported an altered feeling of the tongue; burning, tingling and numbing sensations. This feeling would come and go, and was mentioned by one participant to have a direct effect on the ability to perceive textures and taste:

*Participant 7: F, 59: “I have days where I feel something burning, and it feel on the outer edge of my tongue. I actually have an almost constant burned tongue sensation.”*

*Participant 12: F, 40: “It is like I have something (points at the middle of her tongue), in the middle, it is like I can only taste something if it is on the edge of my tongue (about her tongue feeling burned).”*

*Participant 19: F, 34: “it feels like there is a layer (on her tongue), then I cannot taste as much. But when I do not feel like that, I can taste everything.”*

#### 3.1.3. Food-Related Pleasure

Many participants mentioned that the hedonic aspect of eating was severely altered due to COVID-19. Specifically, the participants expressed a reduced food-related pleasure during the post-acute phase, and eating was, by most participants, expressed to be motivated by a bodily need for energy and nutrients rather than a mental desire.

*Participant 3: F, 29: “If I am a little hungry, I wait (until she become hungrier) as it does not give any feeling of pleasure. Before it was nice to eat, but now I eat to not collapse.”*

*Participant 15: F, 33: “The only thing that matters is to go from being hungry to be satiated. Then you do not have to eat any more. It is not fun or cheering to eat anymore.”*

*Participant 13: F, 43: “Eating is not only for the health of the body. Eating is also for pleasure, and I absolutely do not feel any pleasure of food right now. It is sheer for survival.”*

*Participant 12: F, 40: “You start smelling food and you start saying, Yummy, this smells wonderful (before COVID-19). I am looking forward to eat. This feeling is gone, and it has really bothered me. (…) It is really sad. There is no fun about cooking anymore.”*

The lack of a hedonic incentive to eat was reported to result in an overall lower food intake.

*Participant 3: F, 29: “I have eaten less, and I also eat less now, because it does not make sense to have a second portion. I stop eating when I am full, because there is no enjoyment in it.”*

*Participant 5: F, 26: “For example, I do not eat an extra portion because it tastes good.”*

*Participant 22: F, 25: “Normally the smell of food makes me hungry, and I cannot do that anymore. I do not enjoy food as much. I still eat what I need when I am hungry. It is just not the same joy around food.”*

Being able to perceive taste, flavour and odours, was pointed out as crucial for eating-related pleasure. Furthermore, several of the participants described that the sensory experience was not only affecting perception of the food product, but also overall quality of life.

*Participant 2: F, 44: “Being able to taste these foods means a lot to me, and it affects me mentally. Food has become my enemy. It has been my best friend for so many years.”*

*Participant 9: F, 50: “It is a serious reduction in quality of life, when you lose your ability to taste and smell.”*

*Participant 20: F, 46: “The satisfaction from cooking and serving a meal is completely gone. That is truly sad. (…) it is really becoming quite a plague to cook, and it is not as nice as it used to be.”*

### 3.2. Strategies for Coping with Changes in Appetite and Maintaining Food Pleasure

#### 3.2.1. A Focus on Well-Functioning Senses

Perception of the food’s sensory properties were found to be closely related to the hedonic value of food. All of the participants who experienced dysgeusia and dysosmia changed their diet, excluding foods that tasted bad, and eating ones that they liked. Furthermore, they focused on the senses where perception was maintained, such as chemesthesis.

During the period in which the participants suffered from ageusia, parageusia, anosmia and parosmia, many participants experienced a greater appreciation of crunchy foods. Crunchy foods gave the participants some pleasure when eating, and though the level of pleasure was not perceived to the same extent as before COVID-19, a focus on the well-functioning sense of touch helped them maintain some pleasure with food intake.

*Participant 6: F, 54: “It was very interesting with nuts in chocolate, they are crunchy, so there is still some satisfaction while eating it, even though it had no taste.”*

*Participant 9: F, 50: “it was really gross to eat soft/smattered foods, when it had no taste... I like it when it is something crunchy. I like a crunchy browned surface on meat.”*

*Participant 14: F, 66: “It is an experience to eat a carrot, because it gives my mouth something to work with. Instead of eating something that you chew in seconds, it takes a bit longer to eat a carrot.”*

*Participant 16: M, 48: “to eat something crunchy, that’s also a way to snack.”*

Additionally, adding a creamy component to alter the mouthfeel was appreciated by a few participants.

*Participant 3: F, 29: “e.g., a salad with different veggies, nuts, seeds and some dressing to get something soft, and then it is important that the vegetables still are a bit hard on the inside.”*

*Participant 12: F, 40: “Normally I never use milk in my coffee, but when someone offer me coffee now (after she got COVID-19), I add milk, so I get a different mouth feeling.”*

Several participants experienced that an increased trigeminal stimulation either via a variation in hot and cooling sensations, or an oral burning sensation made them appreciate food more, as it would compensate for ageusia and anosmia. When suffering from these conditions, the participants described that adding chili to a meal, would increase the hedonic experience.

*Participant 3: F, 29: “e.g., a salad with different veggies, nuts, seeds, hot chicken so I get something hot and cold.”*

*Participant 5: F, 26: “Now it is just the flavours and smells (that are missing). (…) Texture, hot food, cold food, a variation, that’s delicious!”*

*Participant 2: F, 44: “The spicier the food, the better I like it. Then I feel like I am getting just a bit of my senses back.”*

*Participant 8: F, 42: “I have tried to eat chili, just because it was fun to feel the burning sensation. I could not taste anything at all.”*

*Participant 12: F, 40: “I always keep a glass of jalapeños in my fridge. I add it to my food just to get some kind of a flavour (to be understood as sensory perception).”*

*Participant 20: F, 46: “It gives a burning sensation. It can also help me get rid of the bad taste.”*

#### 3.2.2. A Focus on Familiarity

Several participants expressed that familiarity of foods became an overly important factor for their hedonic food experience. The memory of how the food used to taste was necessary for their desire to eat and liking of foods.

*Participant 7: F, 59: “One thing I have learned, is that I need to be able to remember how it taste, otherwise I will not like it.”*

*Participant 13: F, 43: “I actually tried eating some spaghetti bolognese from a restaurant the other day. (…) It had no taste at all, but because I know that I used to like it, it still did something for me.”*

*Participant 10: F, 35: “hash (=Biksemad in Danish, a mixed dish with potatoes, meat, vegetables) was on sale, and I thought, we will take it, because I know how it is supposed to taste. That works for me because we have eaten that before, when I was able to taste.”*

*Participant 13: F, 43: “I cannot really taste it, but the feeling of remembering that this is good, sometimes makes me go: Mmmmh Nice! This is really good.”*

Several participants expressed that exposure to unfamiliar food or unexpected sensory stimuli resulted in extreme dislike.

*Participant 7: F, 59: “Just a small thing, a different sound, different look, or different taste, then I instantly loose the desire to eat, and then I give up.”*

*Participant 10: F, 35: “She then picked the wrong one, so I got sausage mix (instead of hash -a dish with potatoes, meat, vegetables) When I tried to eat it, it grew 10 times bigger (in my mouth) than it really was.”*

#### 3.2.3. A Focus on the Eating Environment

In nearly all cases, the participants experienced an increased appreciation of eating with family, as it caused them to shift their focus from not being able to perceive the food’s flavour to enjoying the social company. Furthermore, the social company helped the participants eat proper meals.

*Participant 7: F, 59: “I enjoy it the most, if I am with others. If I am eating alone, I have to convince myself that I have to eat.”*

*Participant 10: F, 35: “But luckily, I cook for myself and my son every day, so the point that I am cooking for someone, and eating with someone—I think that has a big impact on me.”*

*Participant 12: F, 40: “It surely helps eating together, right? I think that was the reason that my appetite has returned. (…) If I had been alone, I would not have eaten anything, so it was really good that I had my kids and my husband.”*

*Participant 15: F, 33: “When I lost my sense of smell and taste, it did not matter anymore—I ate because Claus, my boyfriend, and I had to. Luckily, he was here, otherwise what I ate would not have mattered to me.”*

*Participant 20: F, 46: “Then I am just eating a piece of bread. I do not get advanced when the others are not home. So, I definitely eat less when I am alone.”*

*Participant 22: F, 25: “I would not be cooking if I was eating alone, then I would just eat a small meal. I just enjoy it more when I am eating with someone, I automatically eat a bit more.”*

Even though most participants enjoyed the company of their family, some participants expressed the importance of a calm eating environment during a meal to be able to focus on the sensory properties of the meal that could vaguely be perceived. The participants felt that noise from people around them, as well as food and non-food-related odours could distract their attention, and as a result lower their appetite.

*Participant 2: F, 44: “When I am eating, I need complete silence. I need to focus on what I am eating and focus my mind on the taste it used to have.”*

*Participant 11: F, 43: “Also because we talk a lot, and my family talks a lot, and my husband and daughter talk loudly, it sometimes makes it difficult to be around, they also have to learn to lower their voices. It can affect my appetite so I have to leave the table.”*

*Participant 13: F, 43: “At some point I had to tell her: you have to wash off your perfume, otherwise I will not be able to sit near you.”*

#### 3.2.4. A Focus on Overall Well-Being

As mentioned above, the sense of taste and sense of smell had a big impact on the pleasure related to food intake. When the participants had lost their sense of taste and sense of smell, they focused on other aspects of consumption that could increase their well-being.

Some participants described how they consciously worked with adjusting expectations towards the eating experience and how this helped them not to feel disappointed.

*Participant 9: F, 50: “It must come back—and if it do not, there is really nothing I can do about it.”*

*Participant 14: F, 66: “If I am expecting to be able to taste the meal, then it is going to feel like a bummer. If I have already decided to focus on the texture, or the looks of it, it is not going to be fantastic, but kind of like what I came for—it is living up to my expectations.”*

*Participant 14: F, 66: “I do not want to be negative about it, It is going to be some long days.”*

*Participant 18: M, 53: “It is what it is, just going to have to wait till things turn around.”*

*Participant 19: F, 34: “So you accept the circumstances (...) Oh well, it is what it is, you just got to wait for it to return.”*

Some participants found pleasure in foods they used to perceive as ‘highly palatable’ and ate according to what they previously liked, despite the inability to perceive the flavour. The highly palatable foods were often unhealthy foods like chocolate and chips. For this group, taste was secondary, and the feeling of giving themselves a treat was primary.

*Participant 6: F, 54: “I have always had kind of a chocolate addiction. I could control it for a couple of months at a time, but would always get a fall back, and regain control after a couple of months. (…) I would typically fall in at Christmas, and regain control around easter. It was the same every year. When we were home sick (with COVID-19), we ate a lot of chocolate.”*

*Participant 16: M, 48: “I ate a lot of chips, pork crisps and chocolate. It got out of hand. The less I was able to taste, the more I ate.”*

*Participant 22: F, 25: “Well, by making some palatable meals (you bring pleasure to the meal), and not just something like a fried fish fillet on rye bread or something. That you make something a bit more delicious—more tasty.”*

Other participants cut down on all unhealthy foods and redirected their pleasure from primarily being driven by the food’s flavour to focusing on the healthiness and the feeling of eating nutrients, which are considered good for their body. This group particularly focused on eating healthier, and cut down on all hyper caloric and unhealthy foods.

*Participant 3: F, 29: “When you cannot tell the difference, you might as well just eat healthy.”*

*Participant 13: F, 43: “I have told myself: if you cannot taste it, there is no reason to fill yourself with sugar, there is no reason for that anymore.”*

*Participant 15: F, 33: “I thought of eating mackerel in tomato with my oatmeal in the morning because I could not taste anything. I did not get to try it though.”*

*Participant 16: M, 48: “And I think, in some way, I have been addicted to taste, because I have significantly changed my eating habits since we found out that when we were not able to taste anything, we might as well hire a dietician to help us eat a bit healthier.”*

## 4. Discussion

Overall, the participants were able to reflect upon and describe how COVID-19 affected their appetite, sensory perception and food-related pleasure. Conversely, participants in general showed difficulties verbalizing the strategies applied. This could be related to patients coping on a subconscious level and thereby not being capable of memorizing their strategies up front when asked in an interview. Via questioning, and especially elaboration of patients’ behaviour during meal preparation and intake, several strategies could be identified and interpreted (see Section 3.2).

### 4.1. COVID-19′s Effect on Sensory Perception

Chemosensory dysfunction is common when suffering from COVID-19 [2,4], and as other studies indicate, the majority of COVID-19 patients suffered from chemosensory dysfunction, either in the acute phase, or the sequelae thereof. Specifically, olfactory and gustatory impairments are common among COVID-19 patients, both when reviewing the literature [27,28], and when looking at the participants of the present study. In the present study, the frequency of a combined olfactory and gustatory loss was more common, than suffering from gustatory or olfactory loss separately, which was also the results of a recent study [28]. Some participants experienced the condition worsening over time, observed as anosmia and ageusia developing into parosmia and parageusia. Whether this development in sensory dysfunction is a symptom of soon recovery or opposing long-term effects, cannot be concluded based on the present study.

### 4.2. COVID-19′s Effect on Appetite

Most participants reported experiencing alterations in appetite sensations, and consequently, food intake, due to COVID-19, but the quality of this change (higher versus lower appetite and food intake) varied between the participants. The majority of participants experienced a decreased appetite and consequently decreased food intake, whereas fewer participants experienced a constant unsatisfied appetite, resulting in a constant search for food and thus increased food intake. These results support results from previous studies on patients with chemosensory disorders, where more patients reported to eat less than eat more [21,29].

#### 4.2.1. Decreased Appetite and Intake

Common flu and common cold often correlate with reduced appetite [30]. Furthermore, a study on patients suffering from chemosensory disorders showed that a sudden onset of anosmia, as is the case with COVID-19, is related to weight loss due to lack of appetite [21]. This was likewise reported in the present study, where the participants experienced a reduced appetite during the acute phase of COVID-19, along with the symptoms fever, headache and throat pains (see Section 2.1). Specifically, participants expressed that hunger sensations in general were absent, prior to the meals, and also that a satiated state was reached faster during a meal compared to before COVID-19. Satiation, defined as a feeling of fullness evolving during a meal [31], is linked to the palatability of the food consumed based on perception of the sensory properties. During the early stages of a meal, an ‘appetiser’ effect had previously been found, as a response to palatable food experiences [32]. In line with this, models of appetite suggest a positive-feedback reward mechanism underlying the sensory enhancement of appetite [33]. In several studies, increases in hunger have been reported in the early stages of a meal as a response to palatable foods [32,34,35]. In addition, palatability has also been liked to significant increases in food intake in general [36,37,38]. At later stages during the meal, hunger sensations decline, and the sensation of satiation takes over. As COVID-19 often causes a dysfunctional sensory perception, the palatability of food is suffering. The subjective reports suggesting a faster satiation during a meal could therefore likely be related to a decreased or omitted early stage positive-feedback on appetite, resulting in a faster decrease of hunger sensations and onset of satiation. Furthermore, sensory stimuli are linked to motivation to initiate feeding [35], via value representation of the rewarding properties of food. The link between sensory stimuli, hedonics and appetite has, for example, been observed via the effect of odours on appetite stimulation, and especially via the focus on congruent foods [39,40,41]. As seen in the results from the present study, most of the participants suffered from ageusia and anosmia, and several of them mentioned specifically how this affected their appetite. The ability to perceive odours when cooking a meal was mentioned by several participants as a factor that, under normal circumstances, would induce appetite, but due to the dysfunctional sensory perception, this motivation to eat was not stimulated.

#### 4.2.2. Increased Appetite and Intake

The second group of participants experienced a conditioned increase in appetite, which was expressed as ‘unpleased senses’, and a constant search for foods to reach satisfaction (see Section 3.1.1). This experience is related to the term ‘sensory satisfaction’, which is regarded as a state of contentment where sensory desires are fulfilled [42,43,44]. Eating is both hedonically and homeostatically driven, and the underlying hypothesis behind ‘sensory satisfaction’ propose that a faster hedonic fulfilment via the food’s sensory properties is associated with a lower desire to continue eating, resulting in faster satiation and potentially a reduced intake [45,46]. When suffering from ageusia and anosmia, the sensory stimulation and resulting hedonic response is decreased, which can explain why these participants’ search for sensory stimulation to experience the food-related pleasure they normally experienced before COVID-19. A sensory satisfying meal has previously been found to result in faster satiation, among subjects not suffering from COVID-19 [42,46]. In these two studies, sensory satisfaction increased by altering the trigeminal stimulation during the meal using cayenne pepper. Subjects exposed to the slightly spicier meal felt significantly more sensory satisfied [42], which resulted in reaching a satiated state significantly faster compared to when they ate the meal without added cayenne pepper. This could indicate that a lack of sensory satisfaction could result in an increased intake. The participants in the present study explicitly expressed their satiated sensation as ‘unsatisfied’, as a result of the inability to perceive taste and flavour. although the participants felt satiated, they kept eating, trying to satisfy their desire for sensory stimulation. Similar results have been found among patients suffering from chemosensory disorders [21]. Patients who ate more reported doing so in an effort to taste the food. Therefore, it can be hypothesized that patients who are able to perceive (or sometimes perceive) flavour sensations are the ones increasing their appetite and intake in order to experience the pleasure from the flavour sensation. Future studies will have to clarify this hypothesis.

Increased appetite, and consequently, increased intake, could further be related to the emotional consequences of COVID-19. Though differences were found in the present study with respect to how chemosensory dysfunction affected the participants’ emotional state, all participants expressed negative emotions related to suffering from dysgeusia and dysosmia. A previous study of the effect of COVID-19 on mental and emotional state focused on the effect of quarantine and social isolation [46], but not explicitly related to sensory perception. The study found frequent reports of mental and emotional implications, such as anxiety, emotional distress, and fear during the COVID-19 pandemic [47,48]. Presence of negative emotions such as anxiety, fear and emotional distress, and lack of positive emotions such as happiness, relaxation and positive mood [49], as well as boredom [50], are all emotional states associated with increased food intake. All of the above-mentioned mental or emotional implications were either mentioned by the participants specifically, or could be interpreted from their statements. Therefore, it cannot be ruled out that the emotional effects of the COVID-19 pandemic in general also cause appetite altering effects.

### 4.3. COVID-19′s Effect on Food-Related Pleasure

Food-related pleasure was mentioned directly or indirectly by all participants. Dysgeusia and dysosmia led to low food-related pleasure, and a lot of the participants expressed being surprised to experience the impact of chemosensory dysfunction on well-being.

A recent study including more than 8000 respondents across 14 countries investigated associations between food, drinks and feeling good [51]. Respondents were instructed to write down the first four words that came to mind when thinking about food and beverages and feeling good. The sensory and hedonic value of food was mentioned by 34% of the respondents, and the sub-category ‘taste good’ was mentioned by 24% of the respondents [51]. This study, combined with the broad range of studies and models focusing on sensory properties and acceptance, e.g., [7,8,9], sensory properties and preference [10], sensory properties and food behaviour, e.g., [11,12,13,14], and liking as a determinant for intake [15] highlight the close relationship between sensory properties and pleasure, and when put in the context of the present study, this explains the huge impact of sensory dysfunction on subjective well-being.

### 4.4. Coping Strategies

A key result from the present study was the identification of four key themes when studying the subjective strategies involved in maintaining appetite and food-related pleasure during the acute and post-acute phase of COVID-19: (1) A focus on well-functioning senses, (2) a focus on familiarity, (3) a focus on the eating environment, and (4) a focus on post-ingestive well-being.

#### 4.4.1. A Focus on Well-Functioning Senses

The sensory food experience is related to the five sensory modalities: sight, smell, hearing, taste and touch. The importance of sensory properties and their cross-modal interactions for appetite and hedonic food appreciation is evident, but little has been done to examine the relative importance of each of the five modalities, respectively, for hedonic food appreciation; are all five modalities of equal importance, or does a single (or several) modality/modalities stand out? The few studies conducted previously all point at ‘taste’ (to be understood as flavour, the combined perception of aroma and taste) as being the most important sensory modality for hedonic food appreciation [52,53,54], indicating that consumers do not pay equal attention to all modalities. The importance of flavour was supported in the present study, but the importance of flavour per se in relation to the other modalities differed among the participants and affected subjective food-related pleasure and appetite.

Participants suffering from dysosmia and dysgeusia but who managed to shift their focus from flavour primarily driving food-related pleasure towards focusing on the sense of touch via perceptions of textures, temperatures and burning sensations, described a higher degree of food-related pleasure than the participants maintaining their focus on (lack of) the taste and odour sensations. Though the participants did not experience the same degree of food-related pleasure, as before COVID-19, a focus on the well-functioning senses, or other areas of the meal that brought pleasure, such as social eating (see Section 3.2.3 and Section 4.4.3), helped the participants to maintain some degree of food-related pleasure, as it would increase the chance for the meal to meet their expectations. Inability to focus on the well-functioning sense of touch resulted in increased frustration regarding food intake, and a feeling of dissatisfaction. In studies on patients suffering from chemosensory disorders, similar changes in food habits have been reported. Often, diets were enriched with hot and spicy food [16,21,55]. Additionally, there was a trend towards choosing foods with a crunchy, crispy, smooth and creamy texture [16,21].

The ability to shift focus toward well-functioning senses can be hypothesized to depend on individual differences. The study by Andersen and colleagues [54] further found that for 42% of consumers, the taste (flavour) was the most important sensory modality for the hedonic food experience. For 19% of the subjects, appearance was most important, and for 18%, odour and texture, respectively, were most important. For only 9% of the subjects was taste the least important modality. Therefore, it might be that the participants in our study who managed to focus on the sense of touch were not primarily driven by the flavour in their appreciation of food.

#### 4.4.2. A Focus on Familiarity

Some participants showed reluctance to eat novel foods, especially while suffering from ageusia. Being unable to perceive any tastes or flavours increased the importance of recognizing the food. Eating unfamiliar foods was associated with disgust, and made the participant incapable of continuing the meal. This reaction can be explained by the evolutionary importance of sensory perception for surviving, where the sensory perception guided humankind to avoid ingesting potential harmful foods [56]. The reaction of disgust could therefore be interpreted as an innate reaction, where unfamiliar food that has not been learned to be safe to ingest is rejected, if the sensory properties cannot be perceived and thereby help to determine, whether the meal is safe to eat [57].

#### 4.4.3. A Focus on the Eating Situation

In the present study, many participants expressed having an increased food intake when partaking in a social eating context, for example eating dinner with their family. The participants described the increased food intake to be caused by the focus of the meal to be shifted away from the food itself, and towards the joy of eating with family. No research explains the effect of eating with others when suffering from chemosensory dysfunction, but as discussed in Section 4.2.2, shifting focus away from the flavour and taste of food, towards other aspects of eating still providing pleasure, could increase the food intake. Eating with family and friends is generally known to increase food intake [58], and under normal circumstances, the availability of food per se when eating in a social context can cause an individual to overindulge [59]. Though none of the participants in the current study overindulged while suffering from chemosensory dysfunction, this phenomenon of social eating could still drive the increased food intake reported in the present study.

#### 4.4.4. A Focus on Post-Ingestive Well-Being

Food-related pleasure (and reward) within the appetite space has primarily focused on wanting and liking of food, prior to or during consumption, respectively. Though it has been argued that food-derived reward also depends on the mental and bodily well-being experienced after eating [44], this element has not been researched until recently [60,61,62]. ‘Post-ingestive food pleasure’ is defined as a subjective conscious sensation of pleasure and joy experienced after eating [62] and is driven by both mental and physical sensations, which can be measured via interoception. Interoception functions as a basis for self-awareness and subjective feelings, and can provide insights into the extended appetite experience.

In the present study, it was found that the participants expressed both mental and physical sensations involved in food-related pleasure, and appetite could be maintained by focusing on these both in the relation to the prior to, during and post eating experience.

Specifically, it was found that some participants found pleasure in eating unhealthy foods like chocolate and chips. For this group, the actual flavour was secondary (as it was in many cases reported to be dysfunctional), and the memory of the flavour and the feeling of giving themselves a treat was primary. Calorie-dense foods have previously been proposed as ‘comfort foods’ [63], and when associated with prior experiences, can hold a nostalgic or sentimental appeal [64], as in the present study. As discussed above, suffering from ageusia can have a devastating effect on an individual’s well-being, and it can be the root of several negative emotions. Among the triggers of comfort food eating is the feeling of negative emotions [65], and/or the intent to remedy negative emotions [66]. Eating palatable food is in general known to release mood-enhancing chemicals in the brain [67], which can explain the desire for unhealthy food reported by some participants. Wagner et al. [63] found no difference in subjects’ (*n* = 100) emotional state after watching an upsetting movie, as long as they were offered something to eat. The subjects were either exposed to their favourite comfort food, popcorn, a neutral snack, or nothing to eat at all [63]. Thereby, comfort eating can be interpreted as a subjective behaviour to relieve negative emotions.

In the current study, another group of participants applied the opposite strategy and focused on healthy eating as a source of pleasure. These participants lowered their expectations for the sensory experience, and focused on the holistic feeling of eating foods, which were considered nutritionally ‘good for their body’. This group of participants specifically stopped eating any type of unnecessary or unhealthy foods, as the hedonic aspect related to the sensory properties could no longer be perceived. Interestingly, this group of people reported a higher food-related pleasure and appetite than the group focusing on pleasure from unhealthy foods. The quantitative element of pleasure and the difference between the participants applying a healthy versus unhealthy eating behavioural strategy can be explained in terms of (dis)confirmed expectations. Satisfaction is experienced either when expectations are confirmed, or when experiencing a positive disconfirmation (positive surprise), whereas dissatisfaction is felt when expectations are negatively disconfirmed (disappointment) [68]. Down-adjustment of expectations towards the sensory experience, can thus cause a higher likelihood of expectation confirmation compared to maintaining a focus on the flavour as it used to be perceived, which is more likely to cause a negative disconfirmation with dysfunctional sensory perception as a result of COVID-19. Healthy eating has many positive effects on the body, both mentally [69] and physically [70], and previous studies have found that healthy eating was associated with post-ingestive food satisfaction [71] and post-ingestive well-being [61].

### 4.5. Suggestions for Future Research

The present research increased our understanding of the subjective experience of changes in appetite, sensory perception and food-related pleasure when suffering from the long-term effects of COVID-19. Of potentially even greater importance, the research highlighted potential strategies for coping with these changes to maintain appetite and food-related pleasure. The findings are of relevance for health professionals (e.g., dieticians) working with patients suffering from COVID-19 and chemosensory disorders. In addition, the findings can potentially be extended to other patient groups showing decreased appetite and anhedonia. Therefore, a future focus should be on how to apply the findings among these patient groups.

Along with the application of the findings, the authors suggest that future research focuses on treatments for restoring taste and odour perception. One potential treatment for odour perception impairment is smell training, where patients sniff odours regularly to relearn them. There is evidence from before the COVID-19 pandemic that smell training can improve smell function in people with such impairments [72], but the effect on smell function among COVID-19 patients suffering from anosmia is unknown. In addition to the study of the effect of smell training, the wider applicability of the training procedure in a COVID-19 situation needs to be addressed. For example, it is relevant to know whether anosmic patients in all phases of the COVID-19 disease can benefit from the training, and how to manage the training without the risk of infecting others.

### 4.6. Limitations

From the findings of the present study, it is evident that the sensory food experience is very important for appetite; both for the motivation to engage in food intake and for the continued consumption during a meal. However, as the participants also expressed other symptoms related COVID-19 disease and long-term effects, we cannot conclude that changes in appetite and food-related pleasure are due to sensory impairments only. To be able to study this directly, it would be desirable to compare groups of COVID-19 patients with and without sensory impairments, respectively, and study effects on appetite and food-related pleasure.

A limitation when stating our findings from this qualitative study is that the outcome was strongly dependent on the replies of the participants. A total of 19 people participated, and the results might therefore not apply to the whole group of people suffering from long-term appetite-related effects of COVID-19. Furthermore, the findings are from subjective reports only, and are not backed-up by objective means.

For a total of five participants in the present study, the diagnosis with COVID-19 relied on a subjective assessment, as they were not eligible for a test through the Danish health care system at the time, when they were in the acute phase of COVID-19. Therefore, they do not hold concrete documentation for a diagnosis with COVID-19. However, all five participants mentioned having suffered from symptoms regarded common for COVID-19 during the acute phase of the disease, and were suffering from the common symptoms of long-term consequences of COVID-19, when they were enrolled in the study.

Finally, as the participants were in the post-acute phase of COVID-19 when enrolled in the study, findings regarding the acute phase rely on the memory of the participants. We know from previous studies that retrospection is not necessarily accurate. Therefore, the results regarding the acute phase of COVID-19 should preferably be confirmed in studies in which subjects are not relying on their memory to provide replies.

## 5. Conclusions

Changes in sensory perception, appetite and food-related pleasure are common, both in the acute and post-acute phases of COVID-19. This qualitative in-depth interview study brought a deeper understanding of the subjective experience of these changes, the effect on eating behaviour, and how to cope with the changes to maintain appetite and pleasure when recovering from COVID-19.

The study provides confirmatory evidence for an effect of COVID-19 on chemosensory functions, which include ageusia, anosmia, and parosmia as the most common during the post-acute phase of COVID-19. Chemosensory function was, via the study of dysfunction, found to be the most influential driver for food-related pleasure.

Though all participants suffered from lowered enjoyment of food-related experiences, this study points at new strategies to maintain appetite and food-related pleasure via a focus on other pleasure giving factors when suffering from chemosensory dysfunction. Four key themes were identified; a focus on well-functioning senses, a focus on familiar foods, a focus on the eating environment, and a focus on post-ingestive well-being. The wider applicability of these strategies could be advantageously applied in future studies focusing on day-to-day living with COVID-19 and diseases likewise affecting sensory perception.

## Figures and Tables

**Table 1:** Participant characteristics.

Characteristics	
Nationality	Danish
*N*_total_	19
Gender; males, females	2 males, 17 females
Age; mean (min–max)	44 (25–66)
COVID-19 diagnose (antibody, swab, assessment)	2, 12, 5
Symptoms most often mentioned during the acute phase of COVID-19 (*n*) *	Fever (*n* = 10); Ageusia (*n* = 7); Fatigue (*n* = 7); Anosmia (*n* = 6), Throat pains (*n* = 5), Headache (*n* = 4); Difficulties breathing (*n* = 4).
Symptoms most often mentioned during the post-acute phase of COVID-19 (*n*) *	Ageusia (*n* = 14); Anosmia (*n* = 13); Fatigue (*n* = 6); Parosmia (*n* = 4); Difficulties breathing (*n* = 4)

* Symptoms reported by four or more participants are included in the list of symptoms.

## Data Availability

The data presented in this study are available on request from the corresponding author. The data are not publicly available due to the qualitative character of the study.

## References

[1] WHO (2021). WHO Coronavirus Disease (Covid-19) Dashboard. https://covid19.who.int/.

[2] Vaira L.A., Hopkins C., Petrocelli M., Lechien J.R., Chiesa-Estomba C.M., Salzano G., Cucurullo M., Salzano F.A., Saussez S., Boscolo-Rizzo P. (2020). Smell and taste recovery in coronavirus disease 2019 patients: A 60-day objective and prospective study. J. Laryngol. Otol..

[3] Hummel T., Landis B.N., Hüttenbrink K.-B. (2012). Smell and taste disorders. GMS Curr. Top. Otorhuinolaryngol. Head Neck Surg..

[4] Tenforde M.W., Kim S.S., Lindsell C.J., Rose E.B., Shapiro N.I., Files D.C., Gibbs K.W., Erickson H.L., Steingrub J.S., Smithline H.A. (2020). Symptom Duration and Risk Factors for Delayed Return to Usual Health Among Outpatients with COVID-19 in a Multistate Health Care Systems Network. Morb. Mortal. Wkly. Rep..

[5] Farhadi S., Ovchinnikov R.S. (2020). The Relationship between Nutrition and Infectious Diseases: A Review. Biomed. Biotechnol. Res. J..

[6] Boesveldt S., de Graaf K. (2017). The Different Role of Smell and Taste for Eating Behavior. Perception.

[7] Harper R., Solms J., Hall R.L. (1981). The nature and importance of individual differences. Criteria of Food Acceptance: How Man Chooses What He Eats.

[8] Land D.G., Williams A.A., Atkins R.K. (1983). What is sensory quality?. Sensory Quality in Food and Beverages: Definition, Measurement and Control.

[9] Tuorila H., MacFie H.J.H. (2007). Sensory perception as a basis of food acceptance and consumption. Consumer-Led Food Product Development.

[10] Khan M.A. (1981). Evaluation of food selection patterns and preferences. Crit. Rev. Food Sci. Nutr..

[11] Cardello A.V., Meiselman H.L., MacFie H.J.H. (1996). The role of human senses in food acceptance. Food Choice, Acceptance and Consumption.

[12] Fürst T., Connors M., Bisogni C., Sobal J., Falk L.W. (1996). Food choice: A conceptual model of the process. Appetite.

[13] Connors M., Bisogni C., Sobal J., Devine C. (2001). Managing values in personal food systems. Appetite.

[14] Köster E.P. (2009). Diversity in the determinants of food choice: A psychological perspective. Food Qual. Prefer..

[15] Drewnowski A., Hann C. (1999). Food preferences and reported frequencies of food consumption as predictors of current diet in young women. Am. J. Clin. Nutr..

[16] Merkonidis C., Grosse F., Ninh T., Hummel C., Haehner A., Hummel T. (2015). Characteristics of chemosensory disorders—Results from a survey. Eur. Arch. Oto-Rhino-Laryngol..

[17] Frasnelli J., Hummel T. (2005). Olfactory dysfunction and daily life. Eur. Arch. Otolaryngol..

[18] Miwa T., Furukawa M., Tsukatani T., Costanzo R.M., DiNardo L.J., Reiter E.R. (2001). Impact of olfactory impairment on quality of life and disability. Arch. Otolaryngol. Head Neck Surg..

[19] Temmel A.F., Quint C., Schickinger-Fischer B., Klimek L., Stoller E., Hummel T. (2002). Characteristics of olfactory disorders in relation to major causes of olfactory loss. Arch. Otolaryngol. Head Neck Surg..

[20] Hummel T., Nordin S. (2005). Olfactory disorders and their consequences for quality of life. Acta Otolaryngol..

[21] Keller A., Malaspina D. (2013). Hidden consequences of olfactory dysfunction: A patient report series. BMC Ear Nose Throat Disord..

[22] DiCicco-Bloom B., Crabtree B.F. (2006). Making Sense of Qualitative Research—The Qualitative Research Interview. Med. Educ..

[23] Yin R.K. (2010). Qualitative Research from Start to Finish.

[24] Smith J.A., Osborn M., Breakwell G.M. (2004). Interpretive Phenomenological Analysis. Doing Social Psychology Research.

[25] Noble H., Heale R. (2019). Triangulation in research, with examples. Evid. Based Nurs..

[26] Braun V., Clarke V. (2006). Using Themantic analysis in psychology. Qual. Res. Psychol..

[27] Rojas-Lechuga M.J., Izquierdo-Domínguez A., Chiesa-Estomba C., Calvo-Henríquez C., Villarreal I.M., Cuesta-Chasco G., Bernal-Sprekelsen M., Mullol J., Alobid I. (2020). Chemosensory dysfunction in COVID-19 out-patients. Eur. Arch. Otorhinolaryngol..

[28] Fjaeldstad A.W. (2020). Prolonged complaints of chemosensory loss after Covid-19. Dan. Med. J..

[29] Aschenbrenner K., Scholze N., Joraschky P., Hummel T. (2008). Gustatory and olfactory sensitivity in patients with anorexia and bulimia in the course of treatment. J. Psychiatry Res..

[30] Eccles R., Eccles R., Weber O. (2009). Mechanisms and symptoms of common cold and flu. Common Cold.

[31] Blundell J., Bellisle F., Blundell J., France B. (2013). Satiation, Satiety and the Control of Food Intake: Theory and Practice.

[32] Yeomans M.R. (1996). Palatability and the Micro-structure of Feeding in Humans: The Appetizer Effect. Appetite.

[33] Reichelt A.C., Westbrook R.F., Morris M.J. (2015). Integrating of rewards signalling and appetite regulation peptide in the control of food-cue responses. Br. J. Pharmacol..

[34] Hill A.J., Magson D., Blundell J.E. (1984). Hunger and palatability: Tracking ratings of subjective experience before, during and after the consumption of preferred and less preferred food. Appetite.

[35] Yeomans M.R., Gray R.W., Mitchell C.J., True S. (1997). Independent Effects of Palatability and Within-meal Pauses on Intake and Appetite Ratings in Human Volunteers. Appetite.

[36] Bobroff E.M., Kissileff H. (1986). Effects of changes in palatability on food intake and the cumulative food intake curve of man. Appetite.

[37] Guy-Grand B., Lenhert V., Doassans M., Bellisle F. (1994). Type of test-meal affects palatability and eating style in humans. Appetite.

[38] Hill S.W. (1974). Eating responses of humans during dinner meals. J. Comp. Physiol. Psychol..

[39] Zoon H.F.A., De Gaaf C., Boesveldt S. (2016). Food odours directs specific appetite. Foods.

[40] Morquecho-Campos P., De Gaaf C., Boesveldt S. (2020). Smelling our appetite? The influence of food odors on congruent appetite, food preferences and intake. Food Qual. Prefer..

[41] Ferriday D., Brunstrom J.M. (2008). How does food-cue exposure lead to larger meal sizes?. Br. J. Nutr..

[42] Andersen B.V., Byrne D.V., Bredie W.L.P., Møller P. (2017). Cayenne pepper in a meal: Effect of oral heat on feelings of appetite, sensory specific desires and well-being. Food Qual. Prefer..

[43] Cornil Y., Chandon P. (2016). Pleasure as a Substitute for Size: How Multisensory Imagery Can Make People Happier with Smaller Food Portions. J. Mark. Res..

[44] Møller P. (2015). Satisfaction, satiation, and food behaviour. Curr. Opin. Food Sci..

[45] Andersen B.V. (2014). Sensory Factors in Food Satisfaction. An Understanding of the Satisfaction Term and a Measurement of Factors Involved in Sensory- and Food Satisfaction. Ph.D. Thesis.

[46] Møller P. (2013). Gastrophysics in the brain and body. Flavour.

[47] Canet-Juric L., Andrés M.L., del Valle M., Lópes-Morales H., Poó F., Galli J.I., Yerro M., Urquijo S. (2020). A Longitudinal Study on the Emotional Impact Cause by the COVID-19 pandemic Quarantine on General Population. Front. Psychol..

[48] Kleinberg B., van der Vegt I., Mozes M. (2020). Measuring Emotions in the Covid-19 Real World Worry Dataset. arXiv.

[49] Devonport T.J., Nicholls W., Fullerton C. (2019). A systematic review of the association between emotions and eating behaviour in normal and overweight adult populations. J. Health Psychol..

[50] Abramson E.E., Stinson S.G. (1977). Boredom and eating in obese and non-obese individuals. Addict. Behav..

[51] Sulmont-Rossé C., Drabek R., Almli V.L., van Zyl H., Silva A.P., Kern M., McEwan J.A., Ares G. (2019). A Cross-cultural perspective of feeling good in the context of foods and beverages. Food Res. Int..

[52] Moskowitz H., Krieger B. (1993). What sensory characteristics drive product quality? An assessment of individual differences. J. Sens. Stud..

[53] Moskowitz H., Krieger B. (1995). The contribution of sensory liking to overall liking: An analysis of sex food categories. Food Qual. Prefer..

[54] Andersen B.V., Brockhoff P.B., Hyldig G. (2019). The importance of liking of appearance, -odour, -taste and -texture in the evaluation of overall liking. A comparison with the evaluation of sensory satisfaction. Appetite.

[55] Aschenbrenner K., Hummel C., Teszmer K., Krone F., Ishimaru T., Seo H.S., Hummel T. (2008). The influence of olfactory loss on dietary behaviors. Laryngoscope.

[56] Reed D.R., Knaapila A. (2010). Genetics of taste and smell: Poisons and Pleasures. Prog. Mol. Biol. Transl. Sci..

[57] Pliner P., Pelchat M., Grabski M. (1993). Reduction of neophobia in humans by exposure to novel foods. Appetite.

[58] Ruddock H.K., Brunstrom J.M., Vartanian L.R., Higgs S. (2019). A systematic review and meta-analysis of the social facilitation of eating. Am. J. Clin. Nutr..

[59] Herman C.P. (2017). The social facilitation of eating or the facilitation of social eating?. J. Eat. Disord..

[60] Duerlund M., Andersen B.V., Byrne D.V. (2019). Dynamic Changes in Post-Ingestive Sensations after Consumption of a Breakfast Meal High in Protein or Carbohydrate. Food.

[61] Duerlund M., Andersen B.V., Grønbeck M.S., Byrne D.V. (2019). Consumer reflections on post-ingestive sensations. A qualitative approach by means of focus group interviews. Appetite.

[62] Duerlund M., Andersen B.V., Wang K., Chan R.C.K., Byrne D.V. (2020). Post-Ingestive Sensations Driving Food Pleasure: A Cross-Cultural Consumer Study Comparing Denmark and China. Foods.

[63] Wagner H.S., Ahlstrom B., Redden J.P., Vickers Z., Mann T. (2014). The myth of comfort food. Health Psychol..

[64] Locher J.L., Yoels W.C., Maurer D., Van Ells J. (2005). Comfort foods: An exploratory journey into the social and emotional significance of food. Food Foodways.

[65] Dubé L., LeBel J.L., Lu J. (2005). Affect asymmetry and comfort food consumption. Physiol. Behav..

[66] Evers C., Marijn Stok F., de Ridder D.T. (2010). Feeding your feelings: Emotion Regulation Strategies and Emotional Eating. Pers. Soc. Psychol. Bull..

[67] Magnen J.L. (1986). Hunger.

[68] Deliza R., Macfie H.J.H. (1996). The generation of sensory expectation by external cues and its effect on sensory perception and hedonic ratings: A review. J. Sens. Stud..

[69] Firth J., Gangwisch J.E., Borsini A., Wootton R.E., Mayer E.A. (2020). Food and mood: How do diet and nutrition affect mental wellbeing?. BMJ.

[70] Ezzati M., Riboli M.D. (2013). Behavioral and dietary risk factors for noncommunicable diseases. N. Engl. J. Med..

[71] Andersen B.V., Hyldig G. (2015). Consumers’ view on determinants to food satisfaction. A qualitative approach. Appetite.

[72] Boesveldt S., Postma E.M., Boak D., Welge-Luessen A., Schöpf V., Mainland J.D., Martens J., Ngai J., Duffy V.B. (2017). Amosmia—A clinical review. Chem. Senses.

